# Incomplete insertion of pedicle screws in a standard construct reduces the fatigue life: A biomechanical analysis

**DOI:** 10.1371/journal.pone.0224699

**Published:** 2019-11-01

**Authors:** Yo-Lun Chu, Chia-Hsien Chen, Fon-Yih Tsuang, Chang-Jung Chiang, Yueh Wu, Yi-Jie Kuo

**Affiliations:** 1 Department of Orthopedic Surgery, Wan Fang Hospital, Taipei Medical University, Taipei, Taiwan; 2 Department of Orthopedics, Shuang Ho Hospital, Taipei Medical University, New Taipei City, Taiwan; 3 Department of Orthopedic Surgery, School of Medicine, College of Medicine, Taipei Medical University, Taipei, Taiwan; 4 Graduate Institute of Biomedical Materials and Tissue Engineering, College of Biomedical Engineering, Taipei Medical University, Taipei, Taiwan; 5 Division of Neurosurgery, Department of Surgery, National Taiwan University Hospital, Taipei, Taiwan; University Magna Graecia of Catanzaro, ITALY

## Abstract

Pedicle screws are commonly used for posterior stabilization of the spine. When used in deformed or degenerated segments, the pedicle screws are often not fully inserted into the bone, but instead the threaded portion is exposed by 1 or 2 threads to accommodate rod placement and ensure alignment between the tulip of the screw and the rod. However, broken pedicle screws have been reported with the use of this method. The aim of this study was to determine how the fatigue life of the screw is affected by not fully inserting the screw into the bone. Spinal constructs were evaluated in accordance with ASTM F1717. The following three screw positions were subjected to compression bending fatigue loading; (i) pedicle screw fully inserted in the test block with no threads exposed (EXP-T0), (ii) pedicle screw inserted with one thread exposed outside the test block (EXP-T1), (iii) pedicle screw inserted with two threads exposed outside the test block (EXP-T2). Corresponding finite element models FEM-T0, FEM-T1 and FEM-T2 were also constructed and subjected to the same axial loading as the experimental groups to analyze the stress distribution in the pedicle screws and rods. The results showed that under a 190 N axial load, the EXP-T0 group survived the full 5 million cycles, the EXP-T1 group failed at 3.7 million cycles on average and the EXP-T2 groups failed at 1.0 million cycles on average, while the fatigue strength of both the EXP-T1 and EXP-T2 groups was 170 N. The constructs failed through fracture of the pedicle screw. In comparison to the FEM-T0 model, the maximum von Mises stress on the pedicle screw in the FEM-T1 and FEM-T2 models increased by 39% and 58%, respectively. In conclusion, this study demonstrated a drastic decrease in the fatigue life of pedicle screws when they are not full inserted into the plastic block.

## Introduction

Traditional rigid pedicle screw-rod systems are widely used in the treatment of spinal diseases as they offer immobilization, stabilization and mechanical support to the adjacent vertebrae as an adjunct to spinal fusion procedures. On average, the lumbar region moves through approximately 3 million cycles per year [1,2], and fatigue fracture of implants is a leading cause of post-operative failure of implanted spines [3–6]. Implant failure can disturb the healing process as the support structure can no longer withstand the loading placed upon it [7]. Given that the natural bone fusion process may take up to 12 months, ideally the implanted support structure should be capable of withstanding loading over this period [8].

Improper device design is the main factor to cause the fatigue failure. However, under the same material properties and device design, fatigue life of pedicle screws is more likely to decrease due to improper selection, orientation or position of the screw [9,10]. When pedicle screws are applied to deformed or degenerated segments, the screws are often not fully inserted into the bone, leaving 1 or 2 threads exposed to accommodate rod placement and avoid impingement. Numerous studies have evaluated the relationship between fatigue life and raw materials, surface modifications, and poly-mechanism [11–13], while others assessed the mechanical performance of posterior fixation constructs through experimental tests or finite element simulations [1,2,9,14–16]. La Barbera et al. [9,17] found that the stress on pedicle screws is related to the unsupported screw length exposed outside the vertebra, and their results showed that an unsupported screw length of 2.3 mm leads to a reduction of 3.2 million cycles in the fatigue strength of the tested implant, corresponding to a predicted increase of 40% in the von Mises stress on the screw neck. However, while ASTM F2706 [18] describes a method for determining the unsupported screw length in an occipital-cervical-thoracic spinal implant, there is no similar definition for unsupported screw length in ASTM F1717 [19] for posterior spinal fixation.

In this study, a commercial spinal implant system was subjected to cyclic loading in accordance with ASTM F1717. The aim was to determine how the fatigue life of the pedicle screws was affected by varying the unsupported screw length.

## Materials and methods

### Mechanical fatigue testing

**Fig 1:**
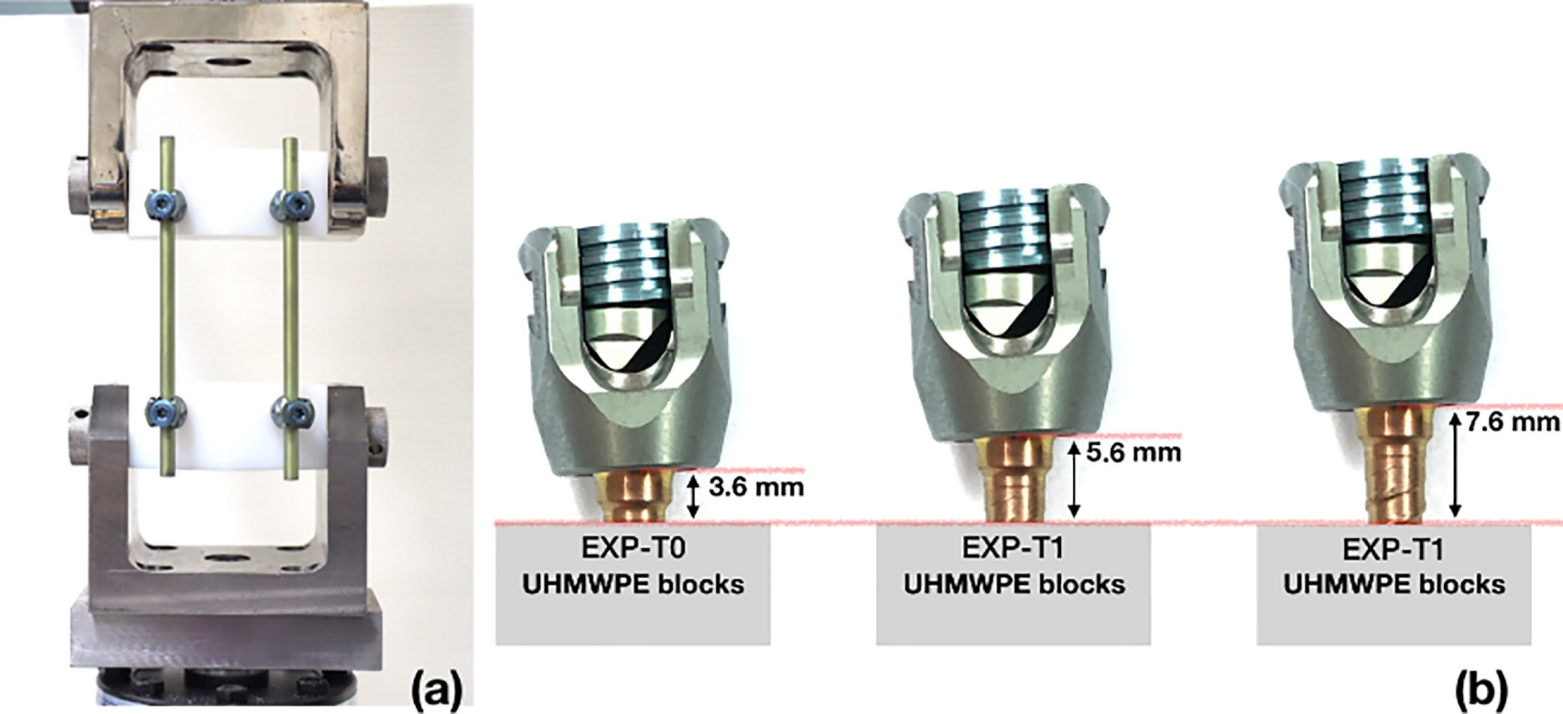
(a) ASTM F1717 standard configuration. (b) Three groups of samples: pedicle screw fully inserted (EXP-T0, unsupported screw length = 3.6 mm), pedicle screw inserted leaving one thread fully exposed outside the block (EXP-T1, unsupported screw length = 5.6 mm), pedicle screw inserted leaving two threads fully exposed outside the block (EXP-T2, unsupported screw length = 7.6 mm).

Fatigue testing was performed using the compression dynamic bending test and loading conditions detailed in ASTM F1717 [19]. A Depuy MATRIX Spine System (Depuy Synthes Spine, Switzerland), which consisted of pedicle screws (Ti-6Al-7Nb, 4.0 mm diameter and 30 mm length) and connection rods (Ti-6Al-7Nb, 5.5 mm diameter and 120 mm length), was placed between custom-made ultra-high molecular weight polyethylene (UHMWPE) test blocks to simulate insertion into a vertebrectomy model (Fig 1A). The screws and rods had their surfaces pre-treated by sandblasting and anodization. The UHMWPE test blocks were then placed between two custom-made side supports and a compressive force was applied via an MTS 370 machine (MTS Systems Corporation, USA). The frequency was set to 5 Hz with a cyclic sine wave. The load ratio value was 10 (minimum load divided by maximum load). ASTM F1717 recommends that loading commence at 50% of the ultimate load, which in this study was determined to be 340 N by performing a preliminary static test on the EXP-T0 construct. Therefore, loading started at 170 N for the EXP-T0 model, and was increased after every third sample until permanent deformation or functional failure occurred, or the number of cycles exceeded 5,000,000 cycles, as recommended by ASTM F1717. Otherwise the load level was decreased every 3 samples until sample run-out. The maximum and minimum loads applied and the number of cycles endured in the fatigue test were recorded to calculate the fatigue strength. Three different setups were evaluated (Fig 1B): (i) pedicle screw fully inserted into the test block without any threads exposed (EXP-T0), (ii) pedicle screw inserted leaving one thread fully exposed (EXP-T1), (iii) pedicle screw inserted leaving two threads fully exposed (EXP-T2).

### Finite element models

**Fig 2:**
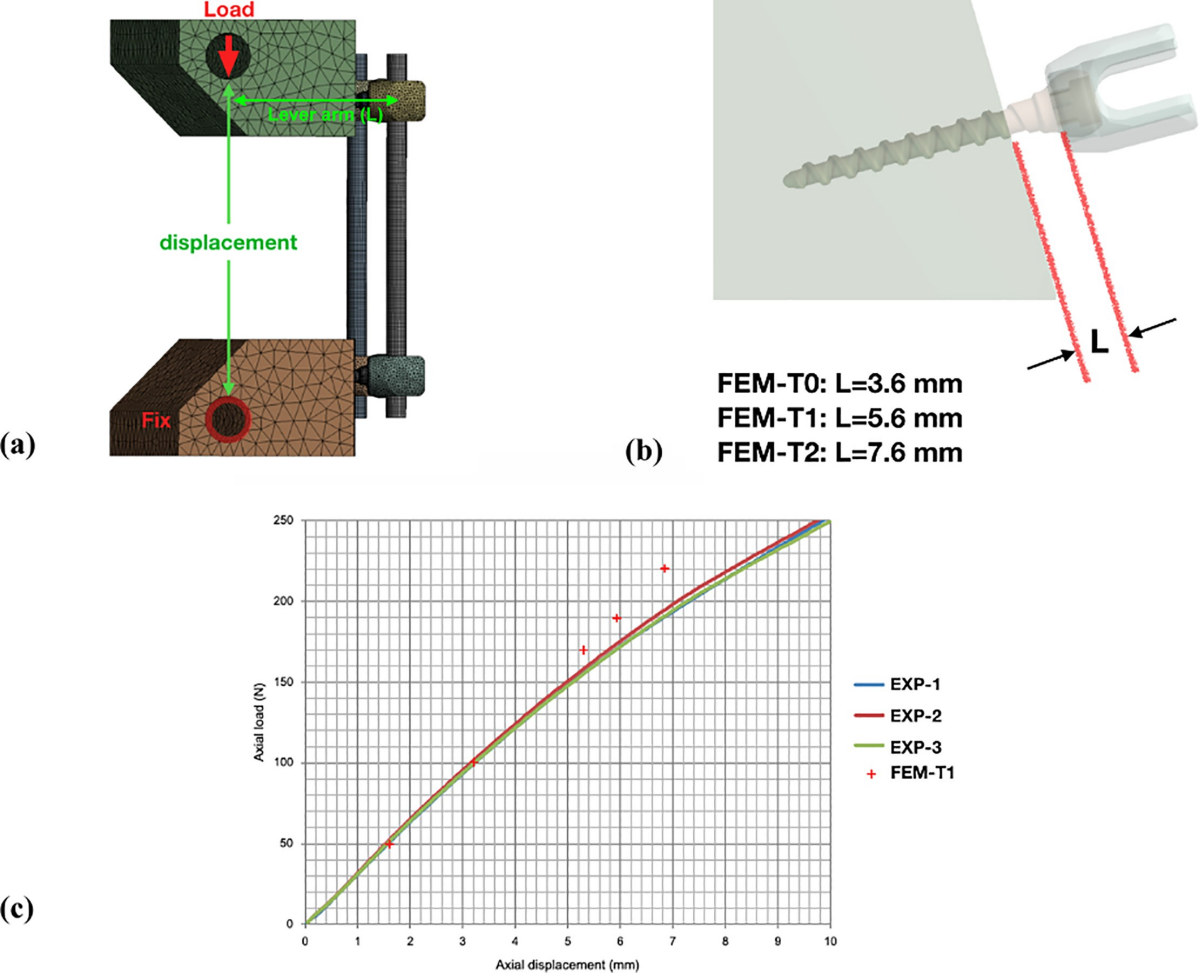
(a) Finite element model in accordance with ASTM F1717 standard configuration. (b) Pedicle screw fully inserted (unsupported screw length = 3.6 mm) (FME-T0), Pedicle screw inserted leaving one thread exposed (unsupported screw length = 5.6) (EXP-T1), Pedicle screw inserted leaving two threads exposed (unsupported screw length = 7.6 mm) (FEM-T2). (c) The axial displacement and load curve of experimental data and finite element models.

Three finite element models (FEM-T0, FEM-T1 and FEM-T2) were created to replicate the experimental fatigue test setup detailed above (Fig 2A and 2B); (i) screw fully inserted (FEM-T0), (ii) one screw thread visible (FEM-T1), and (iii) two screw threads visible (FEM-T2). Similarly, the FE models used the same boundary and loading conditions as the experimental setup. The pedicle screws, rods and UHMWPE test blocks were assumed to be linearly elastic. The material properties of the all components were assigned from literature, as detailed in Table 1. The rod component was discretized using eight-node hexahedral elements, and the screw tulip and body components were meshed using a four-node tetrahedral mesh. The interfaces at the screw-to-rod and screw-to-block were bonded [20,21]. Vertical load was applied to an analytically rigid surface inserted within the horizontal hole of the UHMWPE test block. This rigid surface was assumed to have a frictionless contact with the test block. ANSYS 16.0 (ANSYS Inc., USA) was used for all meshing and simulations. The von Mises stress on the screws and rods, and the stiffness of each test block were recorded. Mesh convergence was determined from the von Mises stress on the screws and on the rods. The convergence criterion used was a change of < 2%, under a loading of 170 N vertical load. The final model had 64,148 elements in each rod, 154,564 elements in each polyaxial screw (34,328 and 150,236 for its head and body, respectively); the element number of each UHMWPE block in FEM-T0, FEM-T1 and FEM-T2 were 61,059, 58,464 and 55,832, respectively.

**Table 1:** Material properties of finite element models.

	Modulus (MPa)	ν	References
UHMWPE blocks	1,050	0.4	(9)
Titanium rods	110,000	0.3	(9)
Titanium pedicle screws	110,000	0.3	(9)

The model was validated by demonstrating that the stiffness of entire model (31.95 N/mm) was within the range of experimental data (31.84~32.48 N/mm) as shown in Fig 2C.

## Results

### Dynamic compression bending test

**Fig 3:**
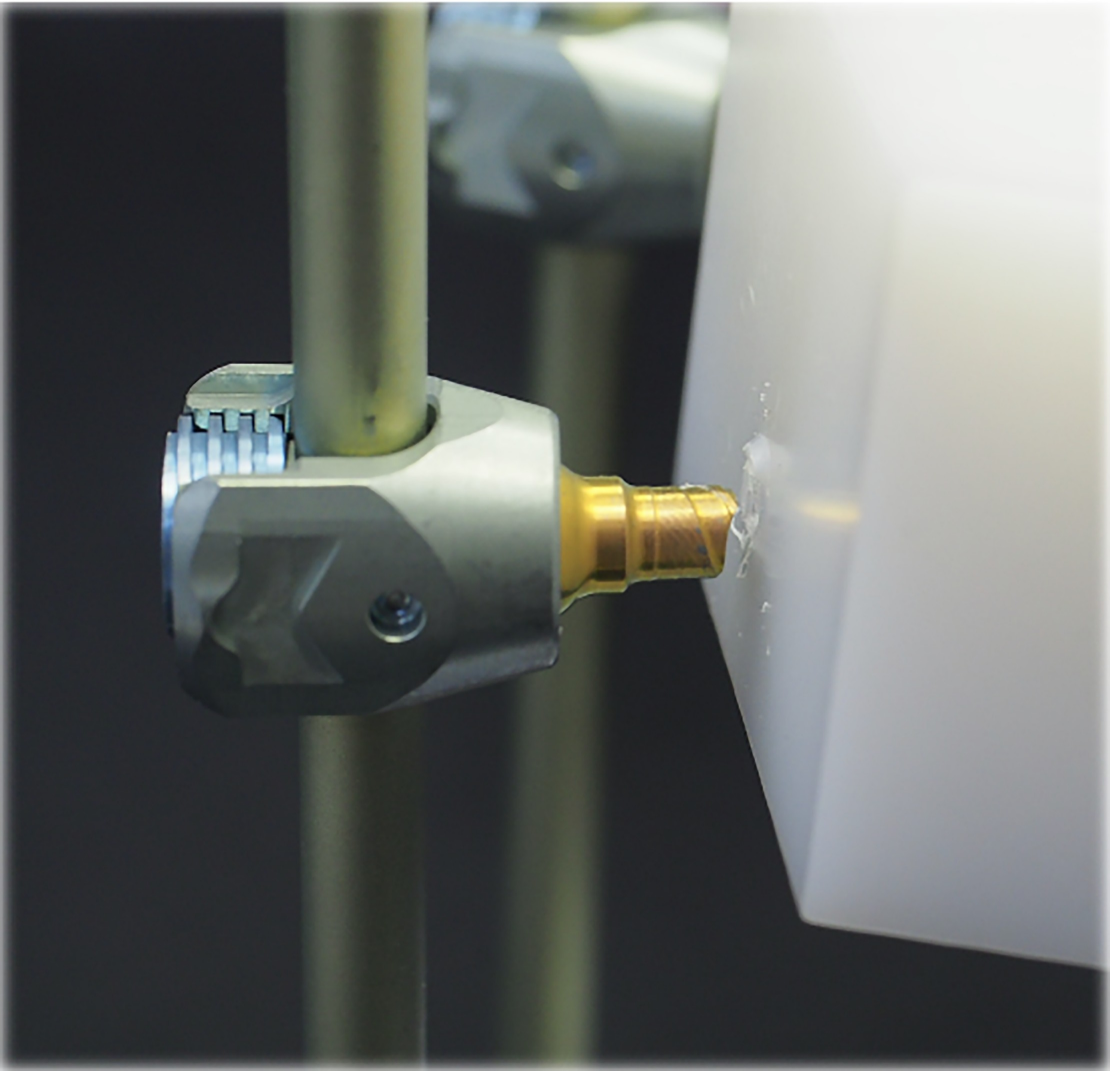
Pedicle screw fracture at the insertion point in the UHMWPE test block.

The results of the dynamic compression bending test showed that the EXP-T0 group had a fatigue strength of 190 N, while both the EXP-T1 and EXP-T2 groups had a fatigue strength of 170 N (Table 2). The implants failed through fracture of the pedicle screw at the point where they entered the UHMWPE block (Fig 3). With a maximum load of 190 N, the EXP-T1 group was able to withstand 3,681,859 cycles on average, which was over 3 times that of the EXP-T2 group (1,032,300 cycles on average).

**Table 2:** Results of dynamic compression bending test.

Min. and Max. of Axial force	17~170 (N)		19~190 (N)		22~220 (N)	
Group	No. of sample	cycles	No. of sample	cycles	No. of sample	cycles
EXP-T0	1	Run-out	10	Run-out	19	697,644*
	2	Run-out	11	Run-out	20	736,998*
	3	Run-out	12	Run-out	21	707,024*
EXP-T1	4	Run-out	13	3,819,921*	22	312,559*
	5	Run-out	14	3,649,685*	23	244,003*
	6	Run-out	15	3,575,971*	24	264,422*
EXP-T2	7	Run-out	16	1,120,864*	25	5,734*
	8	Run-out	17	989,984*	26	9,930*
	9	Run-out	18	986,053*	27	10,659*

*Pedicle screw fracture; Run-out: run out at 5 million cycles which recommended by ASTM F1717.

### Maximum von Mises stress on pedicle screw and rod

**Fig 4:**
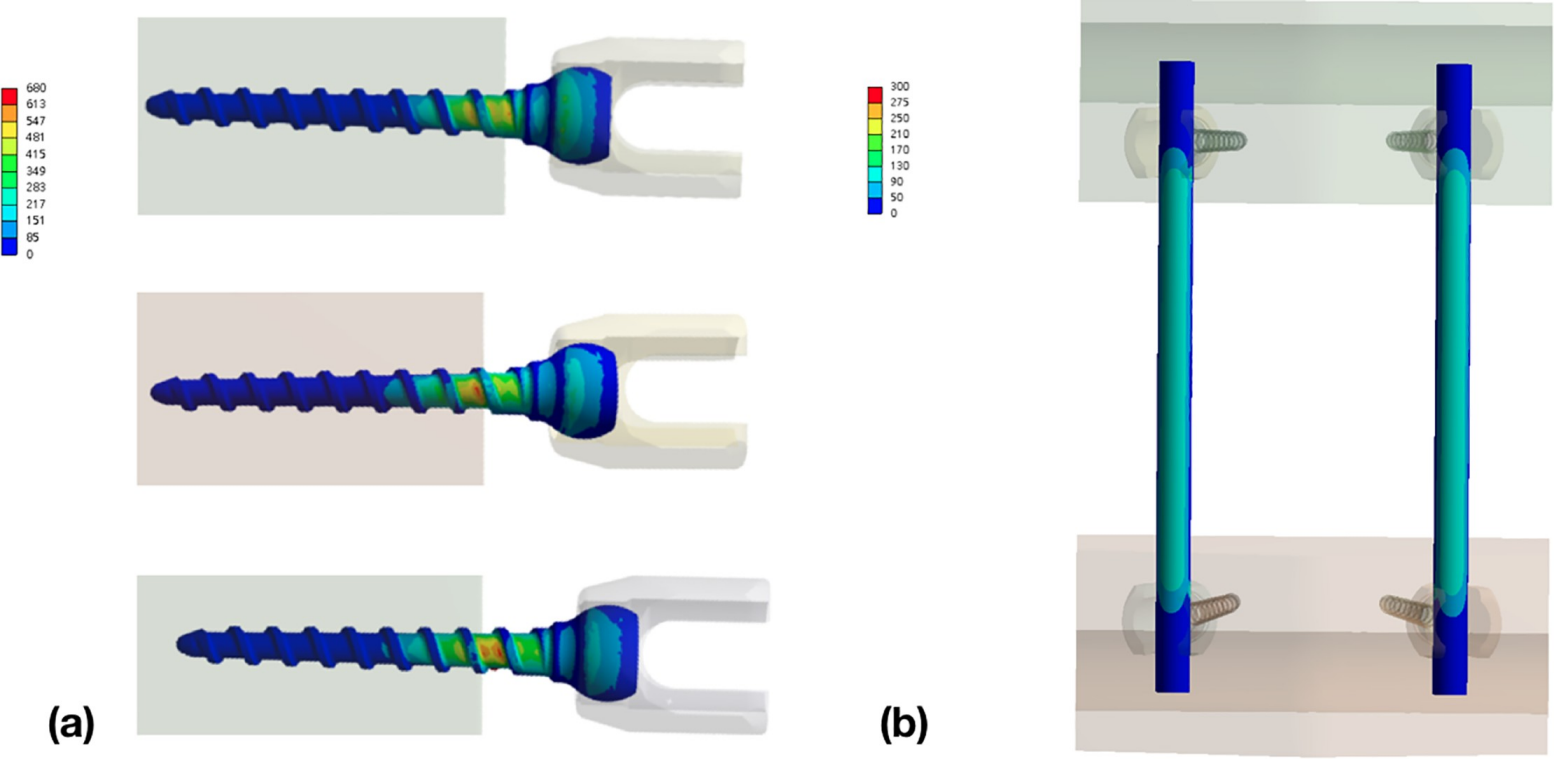
The distribution of maximum von Mises stress in (a) pedicle screws and (b) rods.

The maximum von Mises stress on the screws appeared at the region where the screws entered the UHMWPE blocks (Fig 4A). In the FEM-T0 model, the von Mises stress on the pedicle screw was 676.99 MPa, 758.23 MPa and 873.32 MPa when axial forces of 170 N, 190 N and 220 N were applied, respectively, which were determined from the results of the dynamic compression test (Table 3). For the rod component in the FEM-T0 model, the maximum von Mises stress was 339.53 MPa, 380.27 MPa and 437.99 MPa under the same loads and the maximum value occurred at the interface between the screw and rod (Fig 4B and Table 4). The maximum von Mises stress on the pedicle screw in the FEM-T1 and FEM-T2 models was 39% and 58% greater than in the FEM-T0 model, while the maximum von Mises stress on the rod component increased by 2% and 8.3%, respectively. The stiffness of the FEM-T1 (31.95 N/mm) and FEM-T2 (26.34 N/mm) models decreased by 26% and 39%, respectively, in comparison to the FEM-T0 model (43.18 N/mm). Compared to the experiments, the stiffness of all FEM models was similar to experimental data (EXP-T0: 43.3±0.42 N/mm, EXP-T1: 31.92±0.46 N/mm, EXP-T2: 26.69±0.63 N/mm).

**Table 3:** Maximum von Mises stress in screws.

Axial force (N)	170	190	220
Screw of FEM-T0 (MPa)	676.99	758.23	873.32
Screw of FEM-T1 (MPa)	941.02	1,053.94	1,213.91
Screw of FEM-T2 (MPa)	1,069.64	1,198.00	1,379.85

**Table 4:** Maximum von Mises stress in rods.

Axial force (N)	170	190	220
Rod of FEM-T0 (MPa)	339.53	380.27	437.99
Rod of FEM-T1 (MPa)	346.32	387.88	446.75
Rod of FEM-T2 (MPa)	367.71	411.83	474.34

## Discussion

The goal of posterior spinal fixation is to reconstruct the compromised columns within a spinal motion segment to provide temporary stabilization until bone fusion is achieved. Failure of posterior spinal fixation typically occurs as a result of high-energy impact injuries or metal fatigue from repetitive spinal movements. The primary source of mechanical stress on the fixation screws is from daily physical activities [22], and these stress are suspected to be the main cause of screw fracture. Pedicle screw breakage greatly reduces the support offered for vertebral body fusion during the healing period and has been reported with an occurrence of 4.8–7.1% [23,24]. The FDAs Total Product Life of Cycle (TPLC) database also identifies screw breakage (product code: MNH, MNI)) as one of the primary causes of implant failure. In a retrieval analysis study [7], 75% of screw breaks were reported to have occurred in the proximal region of the screw, with the most common fracture sites being the first or second thread from the screw head. However, this current study demonstrated that when the length of the unsupported screw was increased, the screw tended to break near the entry point in the UHMWPE test block.

The fatigue strength of both the EXP-T1 and EXP-T2 constructs was found to be less than EXP-T0, but all constructs failed in the same manner, through breakage of the pedicle screw. These results were consistent with the FEM calculations. For all FEM models, the maximum von Mises stress on the screw was greater than on the rod, which is consistent with literature [7,9,14–16]. La Barbera et al. [9] demonstrated that decreasing the unsupported screw length could reduce the stress on the screw head. Similarly, in another study, La Barbera et al. [17] reported that when the unsupported screw length was increased from 0 mm to 2.3 mm, the fatigue strength of the screw decreased and the stress on the screw head increased by 40%. In this current study, leaving two threads of the screw exposed (unsupported screw length of 7.6 mm) increased the von Mises stress on the caudal side by 58%. This may explain why screws are often reported to break on the proximal side [7,25]. The experimental setup in this study is similar to La Barbera et al.[17], but not completely consistent. In this study, the unsupported screw length is including the exposed thread except for the baseline model (EXP-T0), and our results reveled the fracture site of screw is near the entry point in the UHMWPE test block; but in the study by La Barbera et al.[17], the range of unsupported screw length is from 0 to 2.3 mm at the screw neck only and their results showed the fracture site is at the screw neck.

Under a 190 N cyclic load, the EXP-T2 constructs failed after approximately 1,000,000 cycles. However, screwing in by a further one thread (leaving one screw thread exposed, EXP-T1 group) allowed the construct to survive up to 3,700,000 cycles, while fully inserting the screw threads (EXP-T0 group with an unsupported screw length of 3.6 mm) allowed the construct to survive the full 5,000,000 cycles. Under a 220 N cyclic load, the average fatigue life of each group decreased drastically in comparison to the groups loaded by 170 N or 190 N, with the EXP-T2 group suffering the greatest decrease. The length of the lever arm (the shortest distance from axial loading axis to screw/rod junction) of the bending moment acting on the screw is a critical factor influencing the fatigue life, with this study showing that the maximum von-Mises stress increased with each incremental increase in the length of the unsupported screw (Table 5).

**Table 5:** Changes in Max. von Mises stress under different length of lever arm.

Model	EXP-T0	EXP-T1	EXP-T2
Length of lever arm (mm)*	L	L+2	L+4
Screw**	100%	139%	158%
Screw neck	100%	103%	107%

*The load direction is perpendicular to the screw in this study.

**the Max. von Mises stress occurred on screw near the entry point in the UHMWPE test block.

The results of this study suggest that increasing the length of the unsupported screw is a potentially dangerous practice and should be avoided if possible. It can greatly reduce the fatigue strength and fatigue life of the screw. Generally, the non-thread part of screw is large enough for polyaxial head movement. Hence, to maintain the fatigue strength of a pedicle screw, it is recommended to fully insert the screw threads into the pedicle. ASTM F1717 does not specify a suitable length for unsupported screws for posterior spinal fixation and, as such, the reduction in fatigue strength may not be a widely recognized risk in clinical settings as well as, in the preclinical evaluation of standard constructs.

There are some limitations to this study that should be noted. The vertebrectomy model used was constructed in accordance with ASTM F1717 and represents a ‘worst case’ condition where the anterior spine is missing and all the load applied on the posterior fixation construct. The complex loading conditions in the human spine have not been fully considered in ASTM F1717 and so the results of this study may not directly predict in vivo performance. ASTM F1717 is typically used to compare different component designs or surgical techniques in terms of the relative mechanical parameters [9,13,26,27]. This study also did not consider variations in screw design and size as the aim was to evaluate the overall effect of partial screw insertion. A simplification in the FE analysis was to model the constructs as linearly elastic homogeneous isotropic bodies and to bond the interfaces between the screw/UHMWPE block, screw/tulip, and tulip/rod. The assumption may cause an overestimation of stress levels and makes the critical loading condition on screws. A single vertical load was also placed on the FE constructs, in accordance with the physical setup, in order to validate the model. Further work may consider expanding the forces placed on the model to more accurately simulate the various stages of gait [22] and consider a standard construct with an anterior support according to ISO 12189 [28].

## Conclusion

The fatigue life and strength of the screw were greatly reduced when the unsupported screw length was increased. In conditions where there is insufficient support for the anterior column, in order to avoid early fracture of the pedicle screw, it is recommended that pedicle screws are always fully inserted into the bone leaving no threads exposed. It is also recommended that manufacturers include appropriate warnings on labelling advising that the fatigue strength of the screws may be reduced if the unsupported length of the screw is increased.
